# Multi-Pig Part Detection and Association with a Fully-Convolutional Network

**DOI:** 10.3390/s19040852

**Published:** 2019-02-19

**Authors:** Eric T. Psota, Mateusz Mittek, Lance C. Pérez, Ty Schmidt, Benny Mote

**Affiliations:** 1 Department of Electrical and Computer Engineering, University of Nebraska–Lincoln, Lincoln, NE 68505, USA; mmittek@gmail.com (M.M.); lperez@unl.edu (L.C.P.); 2 Department of Animal Science, University of Nebraska–Lincoln, Lincoln, NE 68588, USA; tschmidt4@unl.edu (T.S.); benny.mote@unl.edu (B.M.)

**Keywords:** computer vision, deep learning, image processing, pose estimation, animal detection, precision livestock

## Abstract

Computer vision systems have the potential to provide automated, non-invasive monitoring of livestock animals, however, the lack of public datasets with well-defined targets and evaluation metrics presents a significant challenge for researchers. Consequently, existing solutions often focus on achieving task-specific objectives using relatively small, private datasets. This work introduces a new dataset and method for instance-level detection of multiple pigs in group-housed environments. The method uses a single fully-convolutional neural network to detect the location and orientation of each animal, where both body part locations and pairwise associations are represented in the image space. Accompanying this method is a new dataset containing 2000 annotated images with 24,842 individually annotated pigs from 17 different locations. The proposed method achieves over 99% precision and over 96% recall when detecting pigs in environments previously seen by the network during training. To evaluate the robustness of the trained network, it is also tested on environments and lighting conditions unseen in the training set, where it achieves 91% precision and 67% recall. The dataset is publicly available for download.

## 1. Introduction

Researchers have established that changes in animal behavior correlate with changes in health [1,2,3], however, the labor-intensive methods used to monitor behaviors do not lend themselves well to modern commercial swine facilities where just two seconds of daily observation per pig is recommended [4]. Because industry caretakers are typically responsible for thousands of pigs, it is nearly impossible for them to thoroughly assess the health and well-being of individuals using manual observation. This limitation is further compounded by the fact that the effectiveness of human visual assessments are inherently limited by both the attention span and subjectivity of observers [5].

By adapting modern technology to commercial operations, precision livestock farming aims to ease the burden on individual caregivers and provide a solution for continuous automated monitoring of individual animals [6,7,8]. Over the past two decades, researchers have approached this problem from a multitude of different angles [9,10]. These include 3D tracking via wearable ultra-wide band (UWB) devices [11,12], GPS motes [13,14], inertial measurement unit (IMU) activity trackers [15,16,17,18], RFID ear tags [19,20,21], and detection-free tracking using depth cameras [22,23].

This work aims to provide a generalizable vision-based solution for animal detection in group-housing environments. While animal tracking methods using radio identification devices directly provide data on individual animals, there are several disadvantages to using wearable methods when compared to video-based approaches [24,25]. Wearables must withstand harsh environments, they require costly installation and maintenance on a per animal basis, and the localization accuracy of both UWB and GPS systems is typically too low to detect animal orientation, activities, and social behaviors. In contrast, video already provides information-rich data that allows humans to identify precisely what each animal is doing at all times. However, converting digital video to meaningful data without human intervention is an ongoing pursuit in the research community. Existing solutions require sophisticated computer vision algorithms and they have begun to rely increasingly on advancements in machine learning and artificial intelligence [26,27].

Computer vision researchers experienced a dramatic shift in 2012 when Krizhevsky et al. demonstrated that a deep convolutional neural network, trained from scratch without any hand-crafted heuristics, could easily outperform all existing methods for image classification [28]. This shift was made possible by efficient implementations of convolutional neural network (CNN) training [29,30], improvements in computational resources via graphics processing units (GPUs) [31], and large (100K+ images) human-annotated datasets like PASCAL [32], MS-COCO [33], and CityScapes [34]. Modern computer vision applications that locate and classify objects in images often leverage established methods like YOLO [35,36], SSD [37], and Faster R-CNN [38,39] in their pipeline. While common tasks like pedestrian detection lend themselves well to pre-trained networks and existing datasets, there still exists unique challenges that require custom datasets and accompanying solutions.

This work introduces a fully convolutional neural network used to identify the location and orientation of multiple group-housed pigs. The target output of the network is an image-space representation of each pig’s body part locations along with a method for associating them to form complete instances. To train the network, a new dataset is used that contains 2000 images with 24,842 uniquely labeled pig instances. The dataset is divided into a training set and a testing set, and the testing set is subdivided into two sets: one with images depicting the same environments as the training set, and another with images of new environments not represented in the training set. This dataset design allows the robustness of detection algorithms to be tested against novel animal presentations and environments. The three key contributions of this work are (1) a fully convolutional instance detection method, (2) a public dataset for training and evaluation, and (3) metrics that can be used to measure the performance of methods that detect both location and orientation.

The remainder of this paper is organized as follows. Section 2 discusses related work in vision-based animal detection and modern deep learning detection methods. Section 3 introduces the proposed method and the design of the fully-convolutional neural network. The dataset, evaluation methodology, and results are presented in Section 4 and Section 4.6 discusses some key insights and additional practical considerations. Finally, concluding remarks are provided in Section 5.

## 2. Background

Visual detection of multiple moving targets with a static camera often begins with segmentation of foreground objects using background subtraction. If sufficient separation between targets exists, traditional computer vision methods like connected components can be used to easily identify unique instances [40]. However, this is hardly the case for group-housed animals that constantly engage each other socially and often prefer to lie in groups to preserve warmth.

Using Otsu’s method [41] to adaptively threshold background from foreground and Kashiha’s ellipse-fitting method [42], Nasirahmadi et al. [43] used computer vision to identify the lying behavior of group-housed pigs as a function of temperature. While their method achieved approximately 95% accuracy when locating pigs in the images, it was only tested on a single environment where the animals had consistent age and appearance, so it is difficult to predict how well the method generalizes. Ahrendt et al. [44] used a continuously updated model and a 5D Mahalanobis distance between foreground and background pixels to isolate and track the targets, however, the authors point out that the method struggles to identify separate instances when animals are moving quickly and/or overlapping one another. Nilsson et al. [45] used a region-based method to segment group-housed pigs using supervised learning, demonstrating accurate results when the pigs are fully visible (i.e., non-occluded) from a top-down perspective.

A popular approach to dealing with the difficulties of background subtraction using color images is to incorporate depth-imaging cameras [22,46,47,48,49,50,51,52,53,54]. However, while foreground extraction is made simple, depth image processing is still faced with the challenge of separating abutting pigs. To address this challenge, Ju et al. [27] introduced a top-down method that first detects bounding boxes around pigs using the YOLO object detector [36]. When YOLO inevitably detects multiple touching pigs with a single bounding box, their method proceeds by separating segmented images (obtained using a Gaussian mixture model) to establish boundaries between instances. While their overall method achieves 92% accuracy, it is limited to three touching pigs and results were obtained within a single environment over a ten minute window, so generalizability is difficult to assess.

Some approaches using the Kinect depth-sensing camera explicitly define the region of interest (pen) as a three-dimensional bounding box to separate background from foreground. Mittek et al. [23] use this approach to track pigs as ellipsoidal collections of points in the foreground. While their method achieves an average of 20 min of continuous tracking without errors, it requires manual initialization and does not introduce a detection method. The method introduced by Matthew et al. [55] uses regional grouping of surface normals to detect individual instances. They do not perform a formal assessment of the detection accuracy, but the resulting tracking algorithm maintains accurate tracking for an average of 22 s without requiring manual initialization.

Beginning in 2014 with the introduction of R-CNN by Girshick et al. [56], visual detection methods have predominantly used deep convolutional neural networks. Furthermore, they generally fall into one of two categories: (1) top-down approaches that define regions of interest before performing subsequent segmentation or keypoint annotation, or (2) bottom-up approaches that directly segment pixels or detect keypoints without explicitly detecting regions of interest. Mask R-CNN [57], for example, is a state-of-the-art top-down approach for performing instance-level object segmentation and keypoint detection. However, because it relies on a priori region proposal, it is inherently unable to separate objects with significant bounding box overlap—a common occurrence among group-housed animals. In contrast, bottom-up detection and classification is directly done per-pixel in the image space [58]. Two notable methods, OpenPose [59] and PersonLab [60], directly identify keypoints and affinity metrics that allow them to be clustered into whole instances.

Bottom-up keypoint detection was adapted to cow tracking by Ardö et al. [26]. They identified a set of landmarks visible from a top-down view to represent each cow’s location and orientation, and trained a fully-convolutional neural network to detect them in the image space. A post-processing network was then used to convert the annotations to per-pixel orientation classification outputs, resulting in 95% accuracy in correctly labeling all cows in a given image. In a follow-up experiment, they applied the previously trained network to a new environment and observed that it only succeeded on 55% of images, indicating that the network was overfitting to a particular environment.

The proposed method introduces a new bottom-up strategy that identifies multiple pig instances in images as a collection of keypoints. Unlike the approach by Ardö et al. [26], this method preserves the association between keypoints and instances, making it possible to evaluate the performance of the method directly as a keypoint detection method. Furthermore, keypoints provide a precise representation of the pose of each animal, making it possible to identify activities and interactions between animals.

## 3. Proposed Method

The goal of the proposed method is to detect the location and orientation of all visible pigs in the pen environment. The first stage of the process aims to find the location of all pertinent body parts, while the second stage aims to associate them with one another to form whole instances. The following two sections (Section 3.1 and Section 3.2) describe the method used to represent parts and associations within the image space. Section 3.3 then presents a method that interprets these image space representations and produces a set of unique animal instances. Finally, Section 3.4 introduces the fully-convolutional network that takes, as input, an image of the pen environment and attempts to produce the image space outputs described in Section 3.1 and Section 3.2.

### 3.1. Representation of Body Part Location

The proposed method assumed that images of the pen environment were captured from a downward-facing camera mounted above the pen. When trying to detect and differentiate multiple animals in a group-housed setting, a top-down view has three distinct advantages over alternative visual perspectives. Firstly, animals are generally nonoccluded from the top-down perspective unless they are crawling over (or lying on top of) one another. Secondly, the size and appearance of animals is consistent from a top-down perspective, making it easier for a system to identify the animals. Thirdly, one can reliably approximate the 3D position of each animal from their projection onto the 2D image plane by assuming a constant height above the pen floor plane. Approximation is often necessary if 3D coordinates are desired and the single-camera system being used lacks the ability to measure depth.

From a top-down perspective, the part of the animal most likely to be visible was the surface of the back. Thus, in order to represent both the position and orientation of each pig, the proposed method used the image-space location of the tail and shoulder belonging to each animal. Assuming there are *N* animals in the pen, the tail and shoulder position of animal n∈{1,…,N} is denoted $t_{n}=\left(x_{t_{n}},y_{t_{n}}\right)$ and $s_{n}=\left(x_{s_{n}},y_{s_{n}}\right)$, respectively. More specifically, “tail” refers to a surface point along the center ridge of the back that is between the left and right ham. The term “shoulder” refers to a surface point along the center ridge of the back between the shoulder blades. The chosen representation also includes the 2D position of the left and right ears, denoted $l_{n}=\left(x_{l_{n}},y_{l_{n}}\right)$ and $r_{n}=\left(x_{r_{n}},y_{r_{n}}\right)$, respectively. While their visibility is not guaranteed, such as when the animal lies on its side or positions its head in a feeder, their locations can be used to approximate the pose of the head and/or assign animals with a unique visual marker in the form of an ear tag.

**Figure 1:**
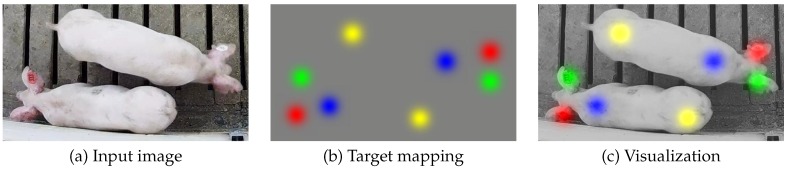
The original image (**a**) is mapped to a four-channel output (**b**), where the location of the left ear, right ear, shoulder, and tail are represented by Gaussian kernels in channels 1–4 of the output, respectively. Note that the four colors used in (**b**) are purely for visualization of the four separate channels of the target image. The overlay in (**c**) is provided to illustrate the locations relative to the original image.

Figure 1a illustrates an example of an input image depicting a single pig. The location of the left ear, right ear, shoulder, and tail are represented by the red, green, blue, and yellow spots in the target mapping shown in Figure 1b, respectively. Finally, the superimposed visualization given in Figure 1c illustrates the locations of the four parts in reference to the original image. Note that the target mapping in Figure 1b has the same spatial dimensions (rows and columns) as the input image, but it contains four channels with each corresponding to a single body part type.

To approximate the level of uncertainty inherent in the user annotations of each body part location, parts within the target mapping were each represented by 2D Gaussian kernels. While the distribution of the 2D Gaussian kernels is defined by a 2 × 2 covariance matrix, here we have only considered symmetric 2D Gaussian kernels that can be characterized by a single standard deviation multiplied by a 2 × 2 identity matrix. This standard deviation will be denoted $\sigma_{n}$ for each animal n∈{1,…,N}. This mapping of uncertainty was meant to approximate the probability distribution of part locations annotated by a human given the original image I. The kernels were also scaled so that the magnitude at the center is 1.0. This allowed for a straightforward translation between kernels and 2D image coordinates via a simple thresholding operation. The first four channels of the target output, as defined in Table 1, were thus proportional to the probability that parts {l,r,s,t} exist at each spatial location in the image.

### 3.2. Representation of Body Part Association

**Figure 2:**
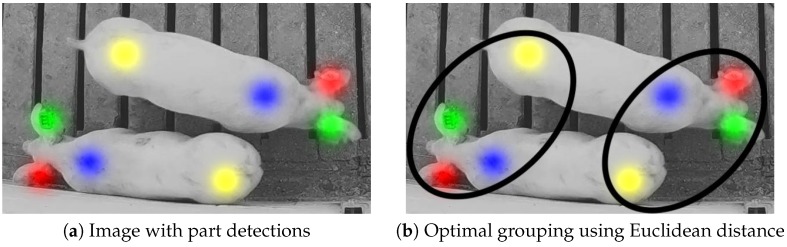
Two nearby pigs with body parts properly annotated are depicted in (**a**). Whole instances must be formed by joining the parts together through part association. An optimal Euclidean nearest-neighbor part association is prone to failure when the animals are in close proximity, as illustrated in (**b**).

Even if every body part location is detected correctly, parts must be associated with each other in order to identify individual whole-animal instances. A naive approach would be to associate each body part with its nearest neighbor in terms of Euclidian distance using an optimal bipartite assignment method, such as the Hungarian algorithm. However, due to the elongated shape of pigs, this approach was prone to failure in cluttered environments, as illustrated in Figure 2.

The proposed method used additional channels in the target output to encode body part locations with 2D vector offsets to other body parts belonging to the same animal. These offsets represented the direction and distance in pixels from one body part to another. While there were a total of 42=6 part pairs that exist between the four parts, the target output only represented three in order to reduce unnecessary redundancy (e.g., vectors joining tail-to-left-ear can be closely approximated by combining a vector joining the tail-to-shoulder and then the shoulder-to-left-ear). Specifically, 12 channels were used to represent three part pair associations, as listed in Table 2. The three part pairs and their associated channels are given below:

- Channels 1–4: Left Ear ↔ Shoulder
- Channels 5–8: Right Ear ↔ Shoulder
- Channels 9–12: Shoulder ↔ Tail

**Figure 3:**
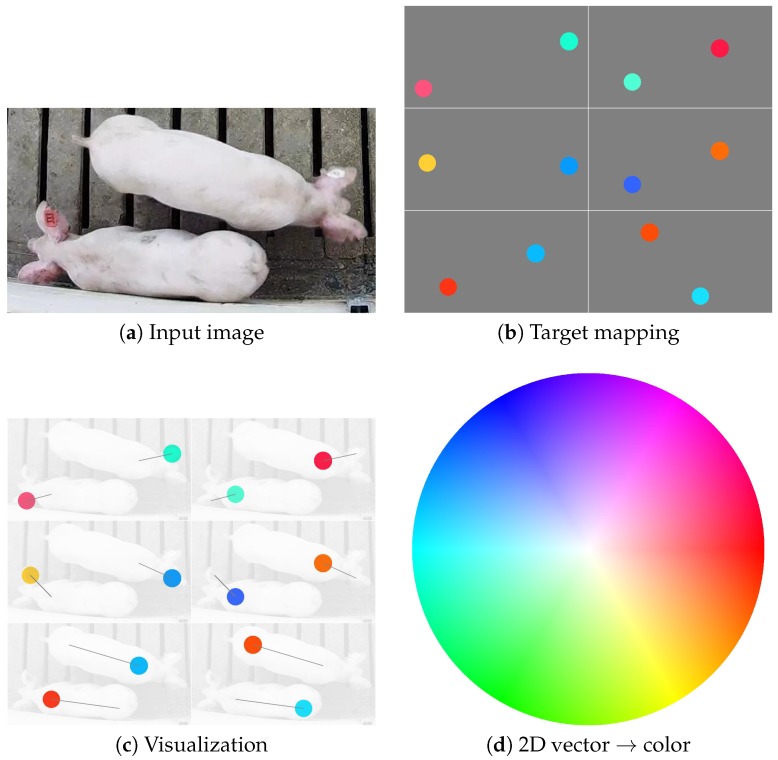
The original image (**a**) is mapped to a 12-channel output (**b**), where vectors joining three pairs of body parts are encoded into circular regions in channels 5–16 of the output. Note that the four colors used in (**b**) are purely for visualization of the direction and magnitude of the vectors, where (**d**) provides a mapping between vectors and colors. The overlay in (**c**) is provided to illustrate the locations of the vector encodings and their magnitude and direction (illustrated by the gray line) in relation to the original image.

Each of the 12 channels encoded a real-valued offset from one point to another. Much like the part detection mappings, these vectors were encoded regionally into the spatial dimensions of the image. Figure 3 illustrates this encoding for a pair of side-by-side pigs. The diameter of the circular regions is denoted $d_{n}$ for each pig *n* in the image, and it is proportional to the standard deviation used for the Gaussian kernel used in the previous section. For visualization purposes, each of the six images in Figure 3b,c represent the direction and distance between part pairs as a color, where the hue represents the direction and the saturation represents the magnitude (encoding provided in Figure 3d). Figure 3c further illustrates the lines joining the parts to one another.

### 3.3. Instances from Part Detection and Association

**Figure 4:**
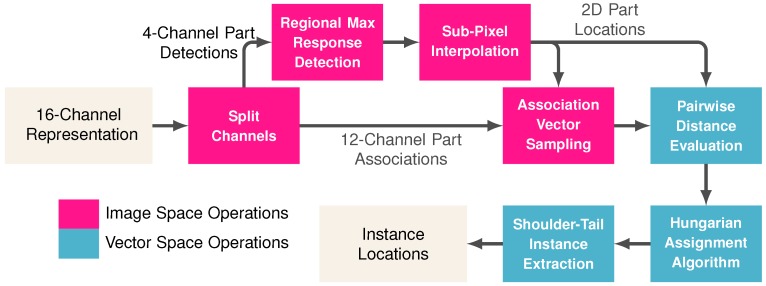
Flow diagram of the proposed method for converting the 16-channel image space representation to a set of 2D coordinates of each visible instance.

The goal of the proposed method was to detect all visible parts and group them together in order to form whole-animal instances. Section 3.1 and Section 3.2 provided a means to represent body part locations and vectors associated them to one another in the form of a 16-dimensional image-space mapping. To translate this image-space mapping to a set of discrete instance locations, one must follow a sequence of steps (illustrated in Figure 4).

First, each 2D body part locations must have been extracted from the Gaussian kernels contained in channels 1–4 of the 16-channel representation image-space representation. Thus, the first step was to split channels into 4-channel part detections and 12-channel part associations. The precise 2D part locations were represented by the peaks of the Gaussian kernels in the image space mapping. Let $M_{p}$ be the R×C image space map for body part p∈{l,r,s,t}, which corresponds with the left ear, right ear, shoulder, and tail, respectively. Note that it was assumed that the number of rows and columns in the input image and output mappings are *R* and *C*. The part locations can be extracted from the image space using a form of regional max response detection defined by

(1) $$\left\{p\right\}=\left\{\left(x,y\right)|M_{p}\left(x,y\right)\geq M_{p}\left(x^{\prime },y^{\prime }\right)\;\mathrm{for}\;\mathrm{all}\;\left(x^{\prime },y^{\prime }\right)\in R_{\left(x,y\right)}\right\}\;\;\mathrm{for}\;p\in \left\{l,r,s,t\right\},$$

where $R_{\left(x,y\right)}$ was a region surrounding image space location (x,y). Part locations are therefore only detected if their value in the image space mapping was greater than that of its neighbors. This worked well for detecting the peak pixel coordinates of Gaussian kernels, but it can be further refined by using quadratic sub-pixel interpolation. Here, interpolation was performed by replacing the original integer coordinates (x,y) with real number coordinates using

(2) $$\left(x,y\right)\leftarrow \left(x+\frac{M_{p}\left(x-1,y\right)-M_{p}\left(x+1,y\right)}{2\left(M_{p}\left(x+1,y\right)+M_{p}\left(x-1,y\right)-2M_{p}\left(x,y\right)\right)},y+\frac{M_{p}\left(x,y-1\right)-M_{p}\left(x,y+1\right)}{2\left(M_{p}\left(x,y+1\right)+M_{p}\left(x,y-1\right)-2M_{p}\left(x,y\right)\right)}\right).$$

Given the complete set of detected body part locations

(3) $$\left\{p_{1},\ldots ,p_{N_{p}}\right\}=\left\{\left(x_{p_{1}},y_{p_{1}}\right),\ldots ,\left(x_{p_{N_{p}}},y_{p_{N_{p}}}\right)\right\}\;\;\mathrm{for}\;p\in \left\{l,r,s,t\right\},$$

the next step was to estimate the locations of associated parts using an association vector sampling of the 12-channel part associations mapping. The 12 dimensions of the association mapping will be denoted $[M_{l\to s}^{x}M_{l\to s}^{y}M_{s\to l}^{x}M_{s\to l}^{y}M_{r\to s}^{x}M_{r\to s}^{y}M_{s\to r}^{x}M_{s\to r}^{y}M_{s\to t}^{x}M_{s\to t}^{y}M_{t\to s}^{x}M_{t\to s}^{y}$] and the estimated location of an associated part q from location $p_{n}$ can be obtained using

(4) $${\left(p\to q\right)}_{n}=\left(x_{p_{n}}-M_{p\to q}^{x}\left(x_{p_{n}},y_{p_{n}}\right),y_{p_{n}}-M_{p\to q}^{y}\left(x_{p_{n}},y_{p_{n}}\right)\right)\;\;\mathrm{for}\;\mathrm{all}\;\;n=1,\ldots ,N_{p}.$$

To join parts together, the distance between the estimated part locations and the actual locations were first computed using a pairwise distance evaluation. Specifically, the association distance between two parts $p_{n}$ and $q_{m}$ is given by

(5) $$d\left(p_{n},q_{m}\right)=\frac{{|\left(p\to q\right)}_{n}-q_{m}{|+|\left(q\to p\right)}_{m}-p_{n}|}{2},$$

where |a| denotes the L2-norm of vector a. Overall, this collection of part-to-part distances forms a set of three unique distance matrices

(6) $$D_{p,q}=\left[\begin{array}{cccc} d(p_{1},q_{1}) & d(p_{1},q_{2}) & \cdots & d(p_{1},q_{N_{q}})\\ d(p_{2},q_{1}) & d(p_{2},q_{2}) & \cdots & d(p_{2},q_{N_{q}})\\ \vdots & \vdots & \ddots & \vdots \\ d(p_{N_{p}},q_{1}) & d(p_{N_{p}},q_{2}) & \cdots & d(p_{N_{p}},q_{N_{q}}) \end{array}\right],$$

where (p=l,q=s), (p=r,q=s), and (p=s,q=t). An optimal assignment between pairs of body parts that minimizes the sum of distances can be obtained by applying the Hungarian assignment algorithm to each distance matrix.

Finally, individual animals are identified as those that contain a joined shoulder and tail. The shoulder-tail instance extraction method began by identifying matches from the Hungarian assignment algorithm’s output for $D_{s,t}$. Once all instances have been identified, the left and right ear detections can be joined to the shoulder locations of all instances via the Hungarian assignment algorithm’s output for $D_{l,s}$ and $D_{r,s}$.

### 3.4. Fully-Convolution Network for Part Detection and Association Mapping

**Figure 5:**
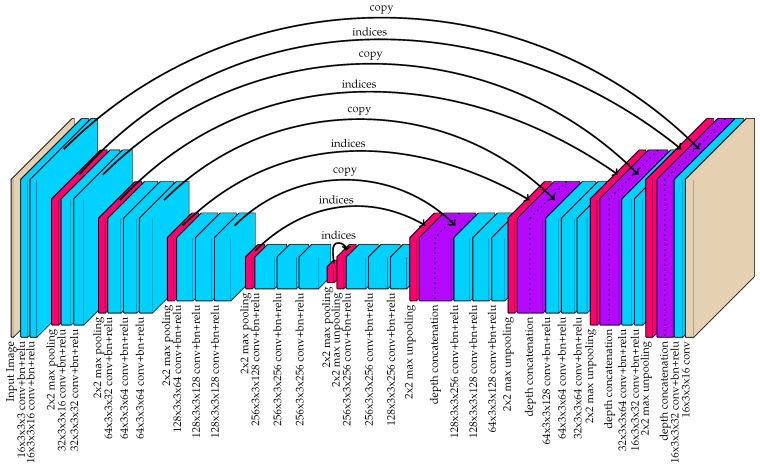
The hourglass-shaped network used by the proposed method to convert images to 16-channel image-space instance detection maps.

A fully-convolutional neural network was used to approximate the 16-channel target output, given a red-blue-green (RGB) image as input. Specifically, the hourglass network illustrated in Figure 5 was proposed. Hourglass networks with symmetry in the downsampling and upsampling stages have previously been used for pose estimation [61] and image segmentation [62]. Specifically, the proposed network was largely based on the popular SegNet architecture [62], which introduced a way to improve upsampling by sharing the indices of each max pooling layer with a corresponding max unpooling layer. This approach has been shown to achieve impressive performance in segmentation tasks by removing the burden of “learning to upsample” from the network.

The network architecture used by the proposed method also incorporated skip-connections in the form of depth concatenation immediately after max unpooling layers. Skip-connections have been demonstrated to encourage feature-reuse, thus improving performance with a fixed number of network coefficients [63]. They were also shown to drastically decrease the amount of training time required by the network. The U-net architecture, introduced by Ronneberger et al. [64], further demonstrated the power of skip-connections for hourglass-shaped networks.

During training, the objective function attempted to minimize the mean-squared error between the network output and the target ground truth. For the first four channels that correspond to part detections, gradients were back-propagated for all pixel locations regardless of their value. For the last 12 channels, gradients were back-propagated exclusively for pixel locations where the target output is assigned (non-zero). Therefore, a specific output was only encouraged in the regions surrounding the point locations. This type of selective training helped to ensure that the vector outputs did not tend toward zero in areas where part detections were uncertain. In essence, this approach was meant to separate the tasks of detection and association mapping.

#### Receptive Field

When designing neural networks for visual tasks, it is important that the network was able to “see” the entirety of the objects it is considering. This viewable area is often referred to as the receptive field. To derive the receptive field, the effective stride length between adjacent coordinates in the feature map is calculated using

(7) $$s_{l_{\mathrm{effective}}}=s_{l-1_{\mathrm{effective}}}\times s_{l},$$

where $s_{l}$ is equal to the stride length at layer block *l* in the network and $s_{0}=1$. Note that, in the proposed network, all max pooling layers have $s_{l}=2$ and all max unpooling layers have $s_{l}=0.5$, while all other layers have $s_{l}=1$. The overall stride length essentially relates the resolution of a downsampled feature map to the original input size. Given $s_{l}$ for all *l* in the network, the receptive field size can be calculated using

(8) $$r_{l}=r_{l-1}+\left(w_{l}-1\right)\times s_{l-1},$$

where $w_{l}$ is the convolutional kernel width at layer *l* and $r_{0}=1$. In the proposed network, each convolutional kernel had width $w_{l}=3$. Because of the stochastic nature of max pooling and max unpooling operations, it was difficult to define their effective kernel size. Therefore, in this analysis, we have used the lower bound of $w_{l}=1$ for all pooling operations.

Table 3 provides the receptive field of the network as a function of layer block for a subset of the 41 layer blocks featured in Figure 5. The receptive field represented the width of a square region in the original image space that affected a single pixel location at the output. While the receptive field of the proposed network was 363, the distance between any two image locations that can affect each others’ outputs was 181 (the radius of the receptive field). In practice, it was recommended that the receptive field size should be considerably larger than the maximum object size due to a decaying effect that has been observed on the outer extremes of the square region [65]. It will be shown in Section 4 that the chosen image scale results in pigs that are typically much smaller than the receptive field radius.

## 4. Experimental Results

### 4.1. Dataset

To the best of our knowledge, no open-source dataset exists for pig detection in group-housing environments. Therefore, to enable quantitative evaluation, a new dataset with 2000 annotated images of pigs was introduced. The dataset (http://psrg.unl.edu/Projects/Details/12-Animal-Tracking) depicts 17 different pen locations and includes pigs ranging in age from 1.5 to 5.5 months. Each unique image was randomly extracted from video recordings spanning multiple weeks in each location. More than two hours, on average, existed between samples at each location. Thus, a wide range of unique animal poses were represented in the dataset.

The dataset was divided into two subsets: 1600 images for training and 400 images for testing. Furthermore, the 400 testing images were subdivided into two additional subsets: 200 captured in the same environments seen in the training set (test:seen), and 200 images from environments previously unseen in the training set (test:unseen). The cameras used to capture the images included both a Microsoft Kinect v2 color camera with resolution 1080 × 1920 and Lorex LNE3162B and LNE4422 color/IR cameras with resolution 1520 × 2688. All of the environments were captured with the camera mounted above the pen looking down. The distance between the pen floor and the camera varied between 2.5 and 6.0 m, and the specific poses of the cameras ensured that the animal pen of interest was centered and entirely contained within the field of view. Variations in environment and image capture technology were used to ensure that the analysis emphasizes robustness.

**Figure 6:**
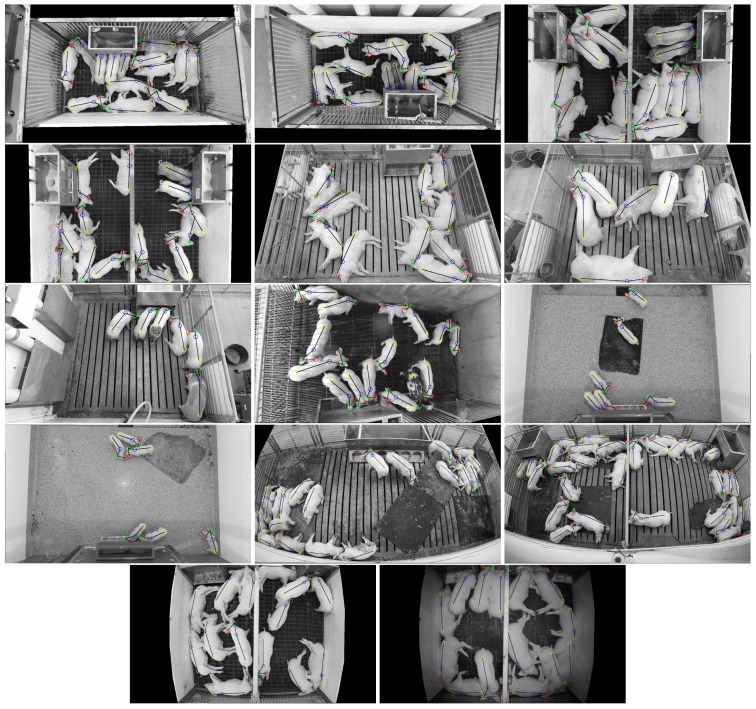
Sample images depicting different environments represented in the training set. The first 13 images (left-to-right, top-to-bottom) were captured during daylight hours with lights on. The last image (from the same environment as the 13th image) was captured using the infrared night vision mode used by the Lorex LNE3162B with active IR illumination.

Figure 6 shows sample images from the training set, depicting 13 different pen locations with color-coded annotations for each hand-labeled body part. Note that the last two images in Figure 6 depict the same environment, but one was captured with full color in the daytime and the other was captured with active IR at night. The first 200 images of the testing set (test:seen) were captured in the same environment as the training set, but at different times. Because more than two hours existed between subsequent randomly sampled images, it is likely that each test:seen image contained different animal poses than each training set image.

**Figure 7:**
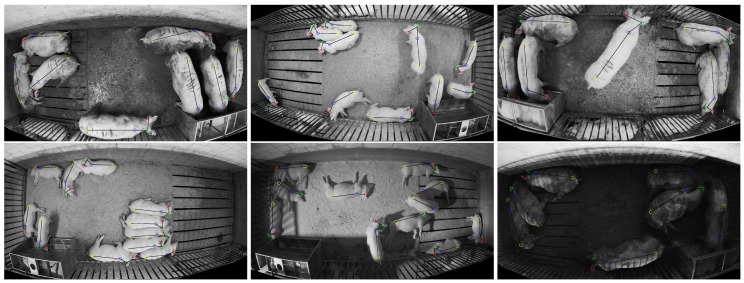
Sample images from the “unseen” portion of the testing set (test:unseen). These images depict environments and lighting conditions not represented in the training set.

Figure 7 illustrates six sample images of the 200 images from the test:unseen set. Note that, not only was this environment previously unseen in the training set, but this set also included challenging lighting conditions that were also not represented among the training images. Twenty images from the training set were captured where the camera’s IR night vision was activated, but all of the remaining 1580 training set images (and all of the test:seen images) were captured with overhead lights on. To achieve the challenging lighting conditions present in the test:unseen set, the lights were turned on at 6 am and off at 6 pm every day. For a short duration between approximately 6 pm and 8 pm, ambient lighting dimly illuminated the pens. After 8 pm, the cameras activated night-vision mode and captured IR images while actively illuminating the scene with built-in IR lights. Two of the four pens presented in the test:unseen set were also illuminated with IR flood lights. This had the effect of creating well lit scenes with harsh shadows and side-lighting.

In each of the images, a user manually annotated the location of the left ear (red), right ear (green), shoulder (blue), and tail (yellow) for each visible animal in that order. Annotations belonging to the same instance are connected with a continuous black line. If ears were not visible, they were not annotated, however, emphasis was placed on annotating both shoulders and tail for each instance even when these locations are occluded, i.e., both shoulder and tail were annotated as long as they are located in the pen of interest and their estimated positions were within the field of view of the camera.

It should be noted that, in some cases, pigs from adjacent pens were partially visible through bars that separate the pens. These partially visible pigs were not annotated. It was assumed that a camera placed above a pen is responsible for detecting only animals in that pen and, while some areas of the image belonging to the adjacent pen were masked out, it was difficult to remove partially visible pigs from the image without unintentionally masking out pigs within the pen of interest. In practice, areas of interests were defined by polygons for each image in the dataset and masking out was done by setting all pixels outside the area of interest to pure black. Examples of masked out regions can been seen in Figure 6 and Figure 7, where the blacked out regions correspond to areas with pigs in adjacent pens.

### 4.2. Training Details

**Figure 8:**
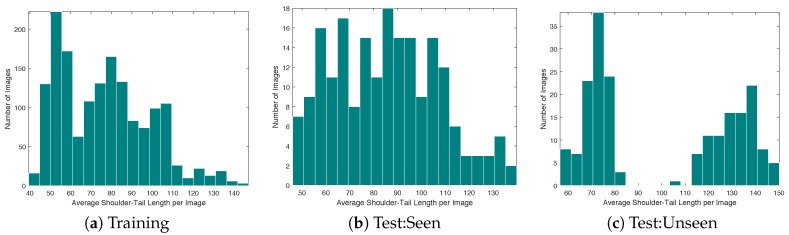
Distribution of the average length from shoulder to tail in each partition of the dataset.

Prior to training the network, images were downsampled so that the number of columns was 480. This was empirically deemed to be a sufficient resolution for discerning the parts of interest while remaining small enough for the computing hardware to process multiple images per second. The average length of pigs in each image after downsampling is presented in the histogram of Figure 8. While the majority of the pigs had a body length of less than 100 pixels, there were some that exceed 140 pixels in length. For these pigs, it was important that the network was able to “see” the entirety of the pig as it estimated the body part locations and vector associations. In Section 3.4, the radius of the receptive field was found to be 181 using the proposed network. Therefore, the network was capable of observing the entire animal even in the most extreme images where the shoulder-to-tail distance approached 140 pixels.

Target images for training the fully-convolutional network were created by adapting the size of the Gaussian kernels used to mark each part in channels 1–4 (Figure 1b) to the size of the animals. This adaptation encouraged continuity of image-space annotations between different environments and ages/sizes of pigs. Specifically, this was done by first computing the average distance between the shoulder and tail for all instances, denoted $\mu_{s\to t}$, to provide a numerical representation of the average size of pigs in the image space. Then, the shoulder-to-tail distance for instance *n*, given by $\delta_{s\to t}$, was combined with the average distance in order to compute the Gaussian kernel standard deviation, defined as $\sigma_{n}=0.16\times \left(\mu_{s\to t}+\delta_{s\to t}\right)$. This combination was used to prevent unusual animal poses from shrinking the size of the kernels too much, while still allowing some adaptation to individual size variations. It should be noted that the scale factor of 0.16 was determined empirically to provide a suitable approximation to the variability of human annotations. If $\sigma_{n}$ was too large, kernels belonging to nearby pigs interfered with each other and often resulted in a single part location being extracted by regional maximum detection. When $\sigma_{n}$ was too small, the network training unfairly applied a penalty to kernels that were not exactly equal to the location provided by the human annotator, even if the kernel’s location was within the natural variance of human annotations. Finally, the Gaussian kernels were then multiplied by a scalar value in order to set their maximum value to 1.0 and, in cases where two nearby Gaussian kernels for the same body part intersected, the output was simply assigned to the maximum of the two Gaussian kernel values. Scaling the kernels so that the peak is 1.0 helped to ensure that a fixed threshold can be used in peak detection regardless of $\sigma_{n}$.

The circular regions used to assign association vectors between parts in channels 5–16 (Figure 3b) should ideally have covered all possible pixel locations where the part might be detected. In practice, this area can be sufficiently covered by considering regions where the Gaussian kernel for each part had a magnitude greater than 0.2. In situations where one region intersected with another, the target output vector was composed of a weighted combination of the two intersecting vectors. The weights in these circumstances came from the corresponding Gaussian kernel magnitude at each pixel location.

The network was trained using heavy augmentation of both input and target images. Augmentations included random left-right flipping, random rotations sampled from a uniform distribution ranging from 0 to 360°, random scaling sampled uniformly between 0.5 and 1.5, and XY shifts uniformly sampled from the range of ±20 pixels along both dimensions. Careful consideration was needed for augmenting the 16-channel target image. Rotations and scaling were applied spatially to both the association vector regions and also the output values along pairs of channels that correspond to XY offsets between body parts. Left-right flips were handled by switching the labels for left and right ears.

### 4.3. Processing Details

After obtaining the 16-channel mapping from the trained network, each of the part location maps (channels 1–4) and the association maps (channels 5–16) were smoothened using a 5×5 averaging box filter. This step would not be necessary to extract information from ground truth mappings, but it was beneficial for reducing the effects of noise on regional maximum response detection. In practice, box filtering was done by adding an average pooling layer to the end of the neural network. The size of regions $R_{\left(x,y\right)}$ used in (1) consisted of a 15×15 window surrounding each pixel (x,y).

The method was implemented in Matlab 2018b using the deep learning toolbox [66]. A desktop computer, equipped with an Intel i7-6700K CPU, 32 GB of RAM, and an NVIDIA GTX1070 GPU was used for training and inference. The approximate time required by the fully-convolutional neural network to perform forward inference is 0.24 s and it took an additional 0.01 s to find instance locations. Thus, the system was capable of fully processing four frames per second.

### 4.4. Instance Detection Performance Metric

The goal of the proposed method was to identify the location and orientation of each pig in a given image. Although the method generated detections and associations for four body parts, only the shoulder and tail location were used to identify a complete instance. This decision was based on two factors. Firstly, they are sufficient for approximating the center-of-mass location and orientation of each animal and, second, special emphasis was placed on ensuring their labeling by human annotators. Given a complete set of *N* ground truth shoulder-tail pairs $\left\{\left(s_{1},t_{1}\right),\ldots ,\left(s_{N},t_{N}\right)\right\}$ and a set of *M* estimated shoulder-tail pairs $\left\{\left({\tilde{s}}_{1},{\tilde{t}}_{1}\right),\ldots ,\left({\tilde{s}}_{N},{\tilde{t}}_{M}\right)\right\}$, an association method was needed to determine if an estimate corresponded to the ground truth, since both sets of pixel coordinates were unlikely to contain exactly the same values.

**Figure 9:**
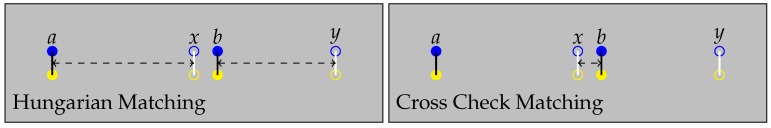
Example with two ground truth instance locations (solid circles connected with black lines) and two detected instances (empty circles connected with white lines) using matching results achieved with both the Hungarian algorithm and the proposed cross-check matching of Equations (9) and (10). While the detection *x* and ground truth location *b* in the middle are clearly nearest neighbors of one another, they are not matched by the Hungarian algorithm. Instead, in an effort to minimize the global matching cost, the Hungarian algorithm will assign *a* to *x* and *b* to *y*. In contrast, the cross-check matching method leaves the outer detection *y* and ground truth location *a* unmatched while assigning the two in the middle, *x* and *b*, together.

Bipartite matching problems are commonly solved with a Hungarian assignment, however, this can sometimes lead to matches between far-away pairs in order to minimize a global cost. For this particular matching problem where shoulder-tail pairs are associated with each other, there was likely to be very little distance between the ground truth and detected positions. Setting the maximum distance allowed between matching pairs can fix this issue, but it comes at the cost of introducing additional parameters that depended on image resolution and the relative sizes of animals. To avoid parameterization, the strict cross-check matching criteria was used here to assign estimates to the ground truth only when they were each others’ minimum cost matches. More formally, two instances *n* and *m* matched if and only if

(9) $$m=\underset{m\in \{1,\ldots ,M\}}{\mathrm{argmax}}|s_{n}-{\tilde{s}}_{m}\left|+\right|t_{n}-{\tilde{t}}_{m}|$$

and

(10) $$n=\underset{n\in \{1,\ldots ,N\}}{\mathrm{argmax}}|s_{n}-{\tilde{s}}_{m}\left|+\right|t_{n}-{\tilde{t}}_{m}|,$$

where || denotes the L2 norm. Figure 9 illustrates the advantage of using the cross-check method instead of the unparameterized Hungarian algorithm.

### 4.5. Instance Matching Results

**Figure 10:**
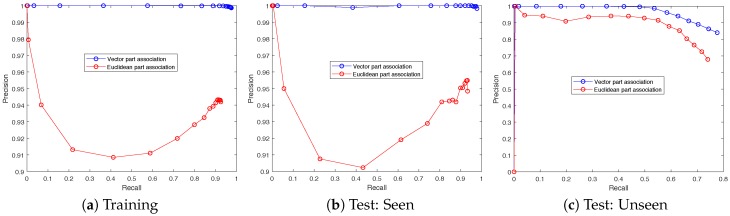
Precision-recall curves for both the proposed method and an alternative association strategy that assigns parts to one another by minimizing Euclidean distance. The results are nearly identical across the training set (**a**) and test:seen set (**b**), and both illustrate a dramatic improvement achieved by joining parts together using the proposed vector mapping. The results on the test:unseen set (**c**) illustrate the limitations of the method when operating on different environments than those used for training.

In order to evaluate the effectiveness of the proposed vector part association method, it was compared to an alternative Euclidean part association method. The Euclidean part association method joins parts together by simply minimizing their Euclidean distance. This method, previously illustrated in Figure 2, removes the effects of part association vectors on detection performance and allows for a partial ablation study. Figure 10 presents the precision and recall for all three partitions of the dataset and Table 4 presents full numerical results over the training set. Each sample along the curve corresponds to a different threshold for part detection, where parts are detected only if the neural network output in channels 1–4 exceeds the threshold value.

The results show that the proposed vector part association method provided a significant boost to matching precision when compared to Euclidean part association. Less than 0.1% of detections were false positives compared to more than 5% when using Euclidean matching, regardless of threshold. Figure 10a,b illustrate nearly identical results across training and test:seen sets. This provides a strong indication that the method was not overfitting to the specific animal poses presented in the training set. Both Figure 10a,b demonstrate a minimum precision of ≈0.91 at a recall of ≈0.42 for the Euclidean matching method. This was because, at this threshold less than half of the animal parts were being detected but the ones that were detected are matched to their nearest neighbor. As a result, there was a relatively high likelihood that only a shoulder is detected, but not a tail, or vice versa. In an effort to form whole instances, the Euclidean method simply joined together nearest neighbors and many of these instances were not aligned with the ground truth. When the threshold was adjusted higher or lower, there was a higher likelihood that either both shoulder and tail were detected or neither was detected. In either case, this lead to improved precision, because the instances that were identified were more likely to be true positives.

In Table 5, the results are compared across all three partitions of the dataset with a fixed threshold of 0.25. While the F-measure was 0.981 at threshold 0.25, which was lower than the peak F-measure of 0.987 achieved at a threshold of 0.1, the decreased threshold produced more than twice the number of false positives. When F-measure values were nearly identical, the choice of threshold depended on how sensitive an application was to false positives and false negatives. The comparison at threshold 0.25 highlighted both the performance similarities across the training and test:seen sets and the discrepancies between both of those sets and the test:unseen set. One interpretation is that the discrepancy illuminates the importance of environment and lighting variations when training the neural network. The test:seen results were nearly identical to the training results, even though the specific poses of each animal were previously unseen. However, due to the use of heavy image augmentation, similar poses were likely represented during training. In contrast, the test:unseen results were much worse, likely due to the novel environments and challenging lighting conditions not present in the training set images.

**Figure 11:**
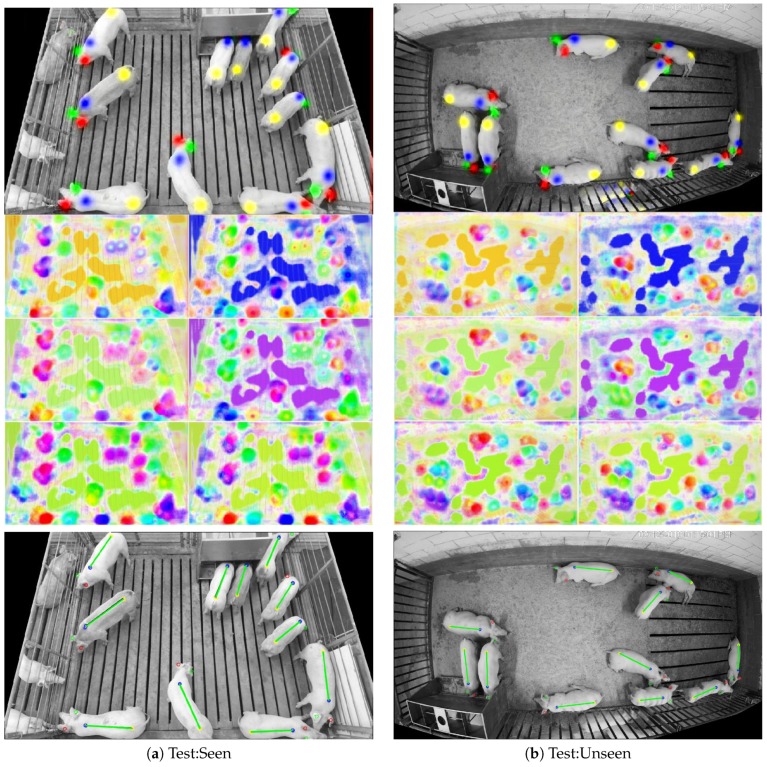
Examples of successful instance detection from both the test:seen set (**a**) and the test:unseen set (**b**). The top images depict the first four channels of the neural network output. The middle image composed of six sub-images depicts the color-coded vector associations from the last 12 channels of the neural network output. The bottom images depict both ground truth locations and estimates using the following color coding: false negative (blue), and true positive (green). Note that these images depict 100% successful detections, so only true positives are present.

By digging deeper into the results and looking at specific examples, it is possible to learn more about the performance discrepancies. An example of 100% successful detections from both test:seen and test:unseen sets are shown in Figure 11. Here, the neural network output is illustrated for each of the 16 channels, and the final detections are shown below, where a green line connecting the shoulder to the tail defines a true positive detection. Note that, unlike the target part association maps illustrated in Figure 3, the outputs of the neural network do not clearly conform to the part locations. This is because the network was only trained to produce the correct vector (illustrated by color) at part locations and, at all other locations, the network essentially tried to minimize the cost with hypothetical part association vectors in case a part was present in that location. This attempt to minimize cost “in case” was most visible when comparing the part association maps of shoulder-to-tail and tail-to-shoulder (the bottom two part association maps in Figure 11). Even when the network was highly confident that a location belonged to a tail, it produced an association vector pointing from shoulder-to-tail at that location, just in case a shoulder at that location was mistaken for a tail.

Due to the similar lighting and overall appearance of test:seen image in Figure 11a, the method was able to identify every instance within the pen environment with high confidence (as indicated by the first four channel outputs of the neural network). However, in the test:unseen image (Figure 11b), the pig behind bars in the adjacent pen caused some confusion in the network. This was likely due to the fact that the network had never been exposed to this particular pen environment, and thus it had not been trained to ignore partial animals on the other side.

**Figure 12:**
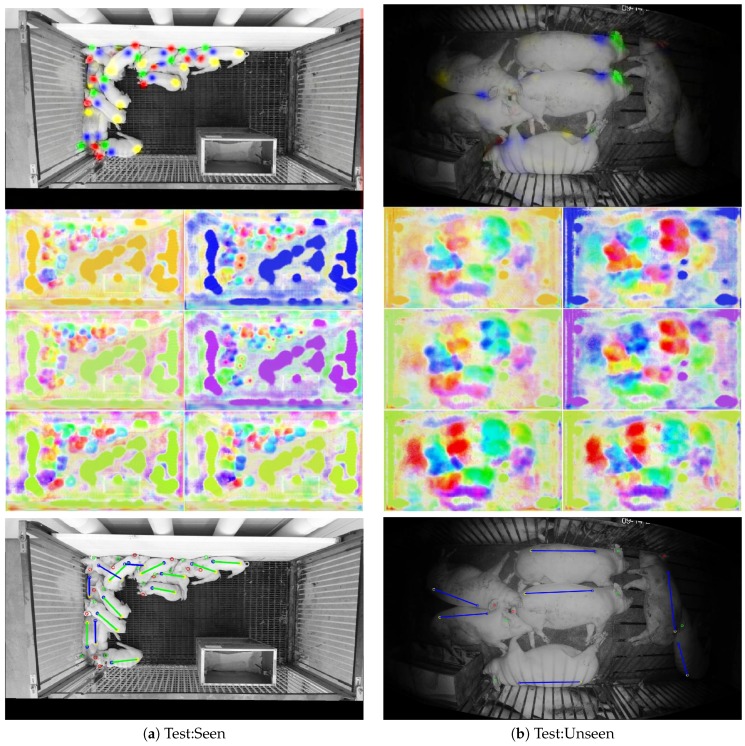
Examples of unsuccessful instance detection from both the test:seen set (**a**) and the test:unseen set (**b**). The top images depict the first four channels of the neural network output. The middle image composed of six sub-images depicts the color-coded vector associations from the last 12 channels of the neural network output. The bottom images depict both ground truth locations and estimates using the following color coding: false negative (blue), and true positive (green).

Alternatively, Figure 12 illustrates failure cases for both test:seen and test:unseen images. Each of the failures in the test:seen image occurred because of occlusions that made it difficult to discern the location of the shoulders and/or tail. In this case, it was even difficult for a human observer to confidently assign the ground truth locations. On the other hand, failures on the test:unseen image were not due to occlusions. They can instead by attributed to the unusual lighting conditions and the relatively large presentation of the animals in the image. Both of these properties were not represented in the training set, making it difficult for the neural network to interpret the image.

**Figure 13:**
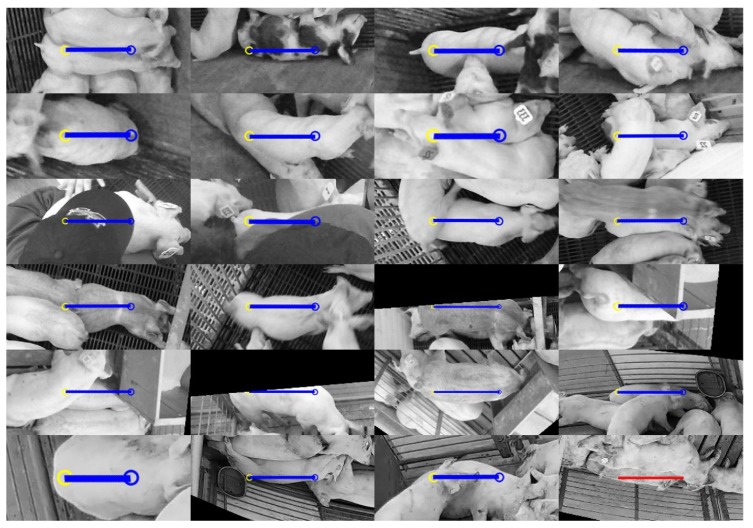
Twenty-four random samples of unsuccessful instance detections from the test:seen set using the following color coding: false negative (blue), and false positive (red).

**Figure 14:**
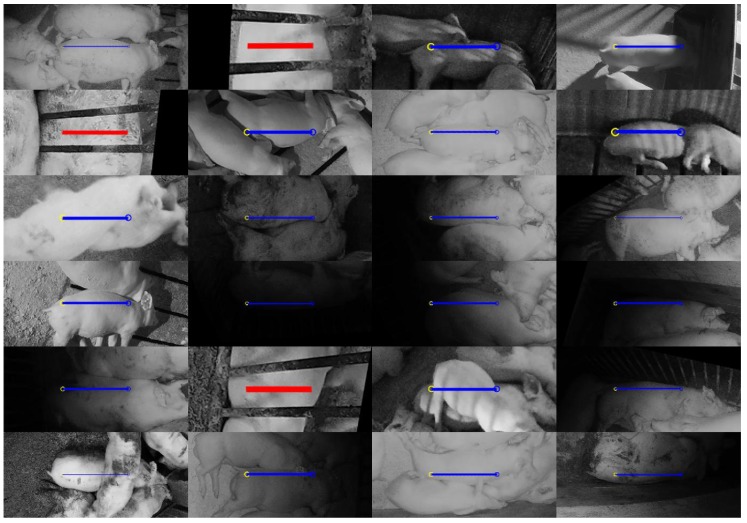
Twenty-four random samples of unsuccessful instance detections from the test:unseen set using the following color coding: false negative (blue), and false positive (red).

Figure 13 and Figure 14 illustrate 24 failures from the test:seen and test:unseen set, respectively. In the test:seen sample set of Figure 13, 17 of the 23 false negatives can be attributed to occlusions or lack of visibility when the pig approached the edge of the image. Some other causes of error include unusual poses where the head was hidden, and situations where the pig had atypical markings. In contrast, only four false negatives out of 21 from the test:unseen sample set (Figure 14) can be attributed to occlusion. At least 10 can likely be attributed to lighting conditions. All three false positives occurred when a pig in an adjacent pen was laying next to the dividing bars. The outline of the bars on the pig’s body appeared to confuse the network into interpreting this as a smaller body pointed in the orthogonal direction.

### 4.6. Discussion

The proposed method focuses on detecting the location and orientation of individual pigs in group-housing environments. Due to the lack of an available public dataset, it was necessary to create a new collection of annotated images. This presented a challenge in terms of capturing the appropriate level of variability and, while we believe that the chosen images sufficiently represent a variety of environments and ages of pigs, it would likely have been beneficial to include more camera angles and more than three types of cameras. Furthermore, the four body part locations were arbitrarily chosen as representatives of the location and orientation of each animal instance. A different set of body parts might have been equally effective.

Compared to datasets like ImageNet [67] and COCO [33], 2000 images may seem like an insufficient number of images to train a deep network. However, pig detection from an overhead camera is a much more specific task than classifying images into one of 1000 categories. With nearly 25,000 different animal poses captured, it is likely that any new pose presented to the network will strongly resemble one that already exists in the dataset. Augmentation is also critical to the success of network training. The chosen network contains nearly 4,000,000 coefficients, so it might be possible to overfit to 25,000 static animal poses, but it is much more difficult to overfit when the angle, size, and left-right orientation is randomized.

The fully-convolutional network introduced in Section 3.4 to estimate body part locations and association vectors was designed with sufficient complexity and a wide enough receptive field to achieve high performance levels in terms of precision and recall. However, the chosen hourglass architecture using max pooling/unpooling with skip connections and depth concatenations is almost certainly one of many network architectures capable of producing the desired 16-dimensional output representation. Atrous convolution, for example has been shown to be particular effective for creating a large depth of field [68] and spatial pyramid pooling has further been demonstrated to achieve excellent performance on multi-scale tasks [69]. A thorough investigation into the suitability of different network architectures might lead to a more accurate or efficient network for instance detection.

By inspecting the specific outputs of the network and the instance formation process, it becomes evident that errors are most commonly caused when the shoulder or tail of one pig occludes the same body part on another pig. Due to the network’s inability to represent multiple part instances in the same image space location, it is only possible for one part instance to be detected in these situations. The vector associations inherently estimate the location of adjacent body parts, therefore, the occlusion can be inferred from the existing output of the network. Alternatively, it might also be possible to augment the dataset to explicitly label occlusions and build the network to detect such events.

In addition to shoulders and tails, the left and right ear were annotated in the dataset and explicitly detected by the network. While the results for instance-level detection do not evaluate the quality of these detections, we foresee them being integrated into future systems as a way to uniquely identify each instance. Ear tags are a common way for livestock to be identified in commercial facilities, and this may provide a convenient way to differentiate between individuals in an otherwise homogeneous population.

Perhaps the most likely application of this detection method would be within a larger tracking system, where detection serves as the first stage for video processing. To this end, the proposed per-frame detection method naturally lends itself to multi-object tracking (MOT). Specifically, a sub-category known as tracking-by-detection MOT methods directly process the outputs of per-frame detection methods, and their performance is often strongly tied to the quality of the detector [70]. For this reason, it is expected that high quality detection methods will eventually contribute to more reliable methods for multi-object tracking.

## 5. Conclusions

The proposed method and accompanying dataset introduced in this paper attempt to provide a robust solution to instance-level detection of multiple pigs in group-housing environments. A major contribution of this work is the introduction of an image space representation of each pig as a collection of body parts along with a method to join parts together to form full instances. The method for estimating the desired image space representation leverages the power of deep learning using a fully-convolutional neural network. Through gradual downsampling and upsampling, the network is able to consider large regions in the image space with a receptive field that covers even the largest pigs in the dataset.

Results demonstrate that the method is capable of achieving over 99% precision and over 95% recall at the task of instance detection when the network is tested and trained under the same environmental conditions. When testing on environments and lighting conditions that the network had not been trained to handle, the results drop significantly to 91% precision and 67% recall. These results can be interpreted in one of three ways: (1) networks should be fine-tuned to handle new environments, (2) a larger number and variety of images should be included in the dataset, or (3) the design and/or training methodology should be revised to improve the robustness to environmental variability. As the dataset and the number of environments grows, eventually there might be enough variety such that new environments add little to the network’s ability to handle novel presentations. Regarding the third interpretation, while significant augmentations were applied to the input and output images during training, it is impossible for spatial transformations to mimic variations in lighting conditions. Therefore, a new set of non-uniform color-space transformations may provide a solution that improves the robustness of the trained network.

By introducing a new dataset and providing an accompanying method for instance-level detection, we hope that this work inspires other researchers to introduce competing methods and perform objective evaluations on the dataset.

## Figures and Tables

**Table 1:** Channels 1–4 of the image-space representation used to represent pig locations and orientations. Each channel corresponds to a different body part. The locations of each part are marked with Gaussian kernels meant to represent the distribution of part locations provided by a human annotator.

Channel	1	2	3	4
Encoding	∝P(l|I)	∝P(r|I)	∝P(s|I)	∝P(t|I)

**Table 2:** Channels 5–16 of the image-space representation used to represent pig locations and orientations. Pairs of neighboring channels corresponds to the *x* and *y* offset between neighboring parts. Overall, these 12 channels represent bidirectional vectors linking three pairs of body parts.

Channel	5	6	7	8	9	10
Encoding	$\propto (x_{l_{n}}-x_{s_{n}})$	$\propto (y_{l_{n}}-y_{s_{n}})$	$\propto (x_{s_{n}}-x_{l_{n}})$	$\propto (y_{s_{n}}-y_{l_{n}})$	$\propto (x_{r_{n}}-x_{s_{n}})$	$\propto (y_{r_{n}}-y_{s_{n}})$
Channel	11	12	13	14	15	16
Encoding	$\propto (x_{s_{n}}-x_{r_{n}})$	$\propto (y_{s_{n}}-y_{r_{n}})$	$\propto (x_{s_{n}}-x_{t_{n}})$	$\propto (y_{s_{n}}-y_{t_{n}})$	$\propto (x_{t_{n}}-x_{s_{n}})$	$\propto (y_{t_{n}}-y_{s_{n}})$

**Table 3:** Sampling of the receptive field calculations at the output of every layer of the proposed network. The different types of layers are abbreviated with the following notation: I: input image, C: convolution block, M: max pooling, U: max unpooling, D: depth concatenation, O: output image.

Layer Type	I	C	C	M	C	⋯	C	M	U	C	⋯	C	U	D	C	O
*l*	1	2	3	4	5	⋯	18	19	20	21	⋯	37	38	39	40	41
$s_{l}$	1	1	1	2	1	⋯	1	2	0.5	1	⋯	1	0.5	1	1	1
$s_{l_{\mathrm{effective}}}$	1	1	1	2	2	⋯	16	32	16	16	⋯	2	1	1	1	1
$w_{l}$	1	3	3	1	3	⋯	3	1	1	3	⋯	3	1	1	3	3
$r_{l}$	1	3	5	5	9	⋯	181	181	181	213	⋯	359	359	359	361	363

**Table 4:** Detailed results obtained with the proposed method and an alternative association strategy that assigns parts to one another by minimizing Euclidean distance. The table includes true positives (TP), false positives (FP), false negatives (FN), precision, recall, and F-measure for different part detection thresholds.

Part Detection Threshold	Vector Matching						Euclidean Matching					
	TP	FP	FN	Recall	Precision	F-Measure	TP	FP	FN	Recall	Precision	F-Measure
0.10	20,217	27	525	0.975	0.999	0.987	19,170	1181	1572	0.924	0.942	0.933
0.15	20,160	20	582	0.972	0.999	0.985	19,127	1156	1615	0.922	0.943	0.932
0.20	20,092	17	650	0.969	0.999	0.984	19,058	1154	1684	0.919	0.943	0.931
0.25	19,999	13	743	0.964	0.999	0.981	18,971	1141	1771	0.915	0.943	0.929
0.30	19,865	10	877	0.958	0.999	0.978	18,851	1135	1891	0.909	0.943	0.926
0.35	19,675	7	1067	0.949	1.000	0.973	18,675	1162	2067	0.900	0.941	0.920
0.40	19,413	3	1329	0.936	1.000	0.967	18,418	1190	2324	0.888	0.939	0.913
0.45	19,029	2	1713	0.917	1.000	0.957	18,077	1195	2665	0.872	0.938	0.904
0.50	18,408	2	2334	0.887	1.000	0.940	17,526	1269	3216	0.845	0.932	0.887
0.55	17,287	2	3455	0.833	1.000	0.909	16,568	1281	4174	0.799	0.928	0.859
0.60	15,227	2	5515	0.734	1.000	0.847	14,871	1294	5871	0.717	0.920	0.806
0.65	11,929	0	8813	0.575	1.000	0.730	12,184	1189	8558	0.587	0.911	0.714
0.70	7565	0	13,177	0.365	1.000	0.534	8543	860	12,199	0.412	0.909	0.567
0.75	3261	0	17,481	0.157	1.000	0.272	4523	430	16,219	0.218	0.913	0.352
0.80	692	0	20,050	0.033	1.000	0.065	1414	90	19,328	0.068	0.940	0.127
0.85	53	0	20,689	0.003	1.000	0.005	143	3	20,599	0.007	0.979	0.014
0.90	1	0	20,741	0.000	1.000	0.000	5	0	20,737	0.000	1.000	0.000

**Table 5:** Instance detection results for the training set (1600 images), the test set with images of the same environments in the training set (200 images), and the test set with images of new environments and challenging lighting conditions (200 images). The part detection threshold was fixed at 0.25.

Evaluation Set	Vector Matching					
	TP	FP	FN	Recall	Precision	F-Measure
Training	19,999	13	743	0.964	0.999	0.981
Test: Seen	2273	1	94	0.960	1.000	0.980
Test: Unseen	1150	112	573	0.667	0.911	0.771
